# Organizing the synaptic junctions

**DOI:** 10.1016/j.jbc.2023.104716

**Published:** 2023-04-13

**Authors:** Qiangjun Zhou

**Affiliations:** Department of Cell and Developmental Biology, Vanderbilt Brain Institute, Center for Structural Biology, Vanderbilt Kennedy Center, Vanderbilt University, Nashville, Tennessee, USA

**Keywords:** synaptic adhesion molecule, *trans*-synaptic, synaptic cleft, MDGA1, global 3D conformation, neuroligin, neurexin

## Abstract

Synaptic adhesion molecules (SAMs) are essential for driving the formation, maturation, and plasticity of synaptic connections for neural networks. MAM domain-containing glycosylphosphatidylinositol anchors (MDGAs) are a type of SAM that regulates the formation of *trans*-synaptic bridges, which are critical for neurotransmission and synaptic differentiation. In a recent issue of the JBC, Lee *et al.* uncovered that MDGA1 can control protein–protein interactions and synaptic cleft activity by adopting different global 3D conformations. This novel molecular mechanism may be applicable to other SAMs that regulate protein–protein interactions and nanoscale organization in the synaptic cleft.

Synapses play a crucial role in facilitating the transmission of nerve impulses between neurons in the nervous system. Abnormal synaptic formation resulting from gene mutations and environmental factors has been implicated in numerous neurodevelopmental disorders (1, 2). Synaptic adhesion molecules (SAMs) are key components that mediate transcellular interactions and help to organize the narrow synaptic cleft, essential for bridging the pre- and post-synaptic compartments (1). In vertebrate brains, SAMs guide synapse formation, drive synapse maturation and differentiation, control synaptic plasticity, and regulate synapse elimination (1). As many SAMs are associated with human neurological and neuropsychiatric disorders, understanding the mechanistic actions of SAMs in regulating distinct properties of various synapse and circuit types is clinically relevant. By understanding the role of SAMs in the nervous system, researchers can gain insights into how the brain works and develop new treatments for neurological disorders.

Several SAMs, including neurexins and MAM domain-containing glycosylphosphatidylinositol anchors (MDGAs), contain multiple repeated domains arranged in a “beads-on-a-string” organization (Fig. 1), similar to typical adhesion molecules. These proteins predominantly act at synapses and play essential roles in the neurotransmission and differentiation of synapses through various mechanisms. Some of these functions require the formation of specific *trans*-synaptic complexes across the synaptic cleft. One such complex is the neuroligin–neurexin complex, where neuroligins are predominantly situated on the postsynaptic side and neurexins on the presynaptic side (3). In particular, the neuroligin extracellular region forms a stable interaction with the specific LNS domain of neurexins. Crystal structures of the neuroligin-MDGA complex have revealed that MDGAs bind to neuroligins on the postsynaptic membrane to block the formation of *trans*-synaptic bridges between neurexins and neuroligins, regulating neurotransmission and synaptic differentiation (4, 5). Another mechanism that significantly contributes to increasing their functional and structural diversity from these multiple repeated domain modular proteins is alternative splicing (1, 6). For example, α- and β-neurexins can be transcribed in many variants, contributing to distinct structural domains and variability. Moreover, splice inserts can regulate the affinities of neuroligins and neurexins for their protein partners, including MDGAs. However, there may be additional mechanisms by which these protein molecules regulate their function. Specifically, the multiple domains of these SAMs can extend to a length of more than 30 nm, which is significantly larger than the width of the synaptic cleft (10–24 nm) (Fig. 1). More research is required to understand if and how these long SAMs adopt and leverage different global 3D conformation and domain arrangements to carry out their function.

**Figure 1:**
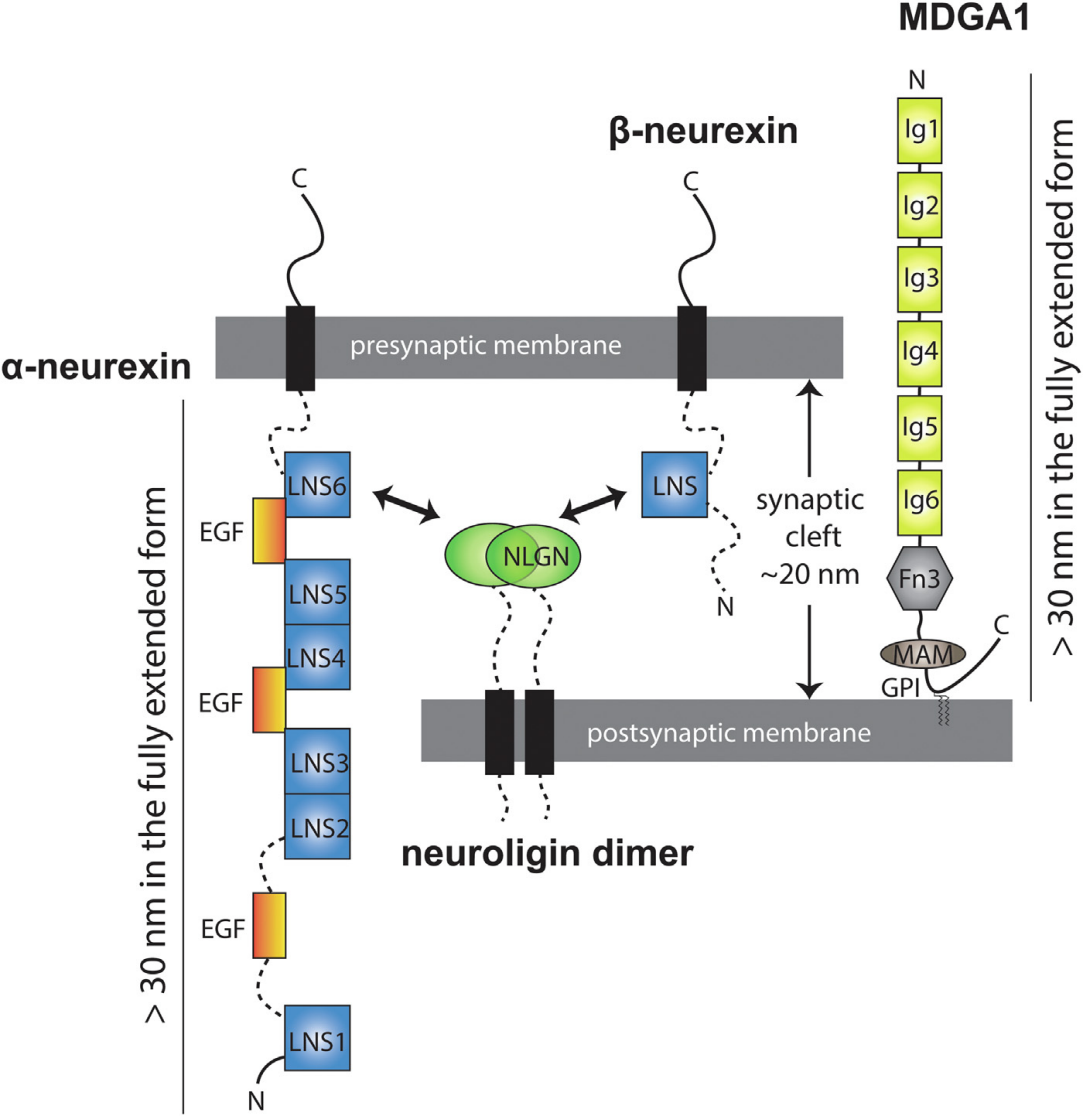
**Domain structures of MDGA1, neurexins and neuroligins.** This figure depicts the proportional representation of the cell membrane thickness, synaptic gap width, and size of each structural domain. The dotted line indicates the possibility of extending a longer loop. All measurements are drawn to scale.

Recently, Lee *et al.* (7) conducted a study to investigate the structure–function relationships of MDGAs by combining a series of biophysical and structural techniques with cell-based assays. Previous studies had revealed that the surface on neuroligins where MDGA1 binds overlaps with the site where neurexins bind, indicating that MDGAs sterically block neurexins from binding to neuroligins (4, 5). Strikingly, the MDGA1 ectodomain (minus the MAM domain) adopts a triangular shape with the 3 sides made up of the domain tandems Ig1/Ig2, Ig3/Ig4, and Ig5/Ig6, respectively. Numerous contacts across the sharply angled Ig2/Ig3 and Ig4/Ig5 elbows appear to stabilize this conformation (4, 5). Initially, the team used biophysical and structural techniques to probe the soluble ectodomain of wild-type MDGA1 (MDGA1 WT) and found that it can adopt both compact (“closed”) and elongated (“open”) forms.

The authors also designed a panel of mutant MDGA1 molecules targeting specific regions to promote either elongated or compact conformations. Their experiments showed that MDGA molecules can take on both compact and elongated conformations, with specific regions working together to produce the overall shape. The team then investigated the ability of MDGA1 WT and mutants to bind neuroligin2 in solution and to block neuroligin2 binding with neurexins in cell-based assays, using both membrane-bound and soluble forms. Most designer mutants in their soluble form did not affect the structure or affinity of the neuroligin2-binding site. However, in their membrane-bound form, many of these mutants lost their ability to bind with neuroligin2 when tethered to the cell surface. The study demonstrated that the global 3D conformation of MDGA1 plays a key role in regulating MDGA1 action within the synaptic cleft.

This work elegantly shows how MDGA1 molecules can adopt different global 3D conformations to regulate protein–protein interactions and could help us further understand the alternative splicing mechanism and the “selectivity code” hypothesis, which states distinct isoforms of MDGA selectively regulate neuroligins for either excitatory or inhibitory synapses, resulting in separate pathways for the formation and maintenance of these two types of synapses (8). Designer mutants can be used as molecular tools to test whether manipulating MDGA1 and MDGA2 can regulate inhibitory and excitatory synapse development/stabilization and engineer unique synaptic connections to improve disrupted neural circuits seen in neuropsychiatric disorders. Further research is needed to determine whether MDGAs dynamically interact with other proteins to form different supercomplexes or nanostructures to carry out various functions. It will also be interesting to see if other multiple-domain SAMs adopt different global 3D conformations to organize nanostructures including *trans*-cellular nanocolumns (9, 10). Finally, it will be exciting to directly visualize the nanoscale organization and remodeling of these SAMs in the synaptic cleft.

In summary, the study by Lee *et al.* proposes a novel molecular mechanism by which MDGA1 regulates protein partner interactions with changes in global 3D conformation. Future studies should investigate the physiological roles of global 3D conformation changes by examining the impact of designer mutants of MDGA1 *in vivo*. Additionally, it will be exciting to see if other SAMs, such as neurexins, share a similar mechanism to regulate protein-protein interactions and nanoscale organization in the synaptic cleft.

## Conflict of interest

The author declares that he has no conflicts of interest with the contents of this article.
